# Etiology of Fever and Associated Outcomes Among Adults Receiving Chemotherapy for the Treatment of Solid Tumors in Uganda

**DOI:** 10.1093/ofid/ofad508

**Published:** 2023-10-12

**Authors:** Elizabeth A Gulleen, Sarah Holte, Yuzheng Zhang, Immaculate Mbarusha, Dennis Mubiru, Bernadette Pedun, Michael Keng, Scott K Heysell, Abrahams Omoding, Christopher C Moore, Warren Phipps

**Affiliations:** Vaccine and Infectious Diseases Division, Fred Hutchinson Cancer Center, Seattle, Washington, USA; Allergy and Infectious Diseases Division, Department of Medicine, University of Washington, Seattle, Washington, USA; Vaccine and Infectious Diseases Division, Fred Hutchinson Cancer Center, Seattle, Washington, USA; Department of Global Health, University of Washington, Seattle, Washington, USA; Vaccine and Infectious Diseases Division, Fred Hutchinson Cancer Center, Seattle, Washington, USA; Uganda Cancer Institute, Kampala, Uganda; Uganda Cancer Institute, Kampala, Uganda; Uganda Cancer Institute, Kampala, Uganda; Division of Oncology, Department of Medicine, University of Virginia, Charlottesville, USA; Division of Infectious Diseases and International Health, Department of Medicine, University of Virginia, Charlottesville, Virginia, USA; Uganda Cancer Institute, Kampala, Uganda; Division of Oncology, Department of Medicine, University of Virginia, Charlottesville, USA; Vaccine and Infectious Diseases Division, Fred Hutchinson Cancer Center, Seattle, Washington, USA; Allergy and Infectious Diseases Division, Department of Medicine, University of Washington, Seattle, Washington, USA

## Abstract

**Background:**

Little is known about the microbiology and outcomes of chemotherapy-associated febrile illness among patients in sub-Saharan Africa. Understanding the microbiology of febrile illness could improve antibiotic selection and infection-related outcomes.

**Methods:**

From September 2019 through June 2022, we prospectively enrolled adult inpatients at the Uganda Cancer Institute who had solid tumors and developed fever within 30 days of receiving chemotherapy. Evaluation included blood cultures, malaria rapid diagnostic tests, and urinary lipoarabinomannan testing for tuberculosis. Serum cryptococcal antigen was evaluated in participants with human immunodeficiency virus (HIV). The primary outcome was the mortality rate 40 days after fever onset, which we estimated using Cox proportional hazards models.

**Results:**

A total of 104 febrile episodes occurred among 99 participants. Thirty febrile episodes (29%) had ≥1 positive microbiologic result. The most frequently identified causes of infection were tuberculosis (19%) and bacteremia (12%). The prevalence of tuberculosis did not differ by HIV status. The 40-day case fatality ratio was 25%. There was no difference in all-cause mortality based on HIV serostatus, presence of neutropenia, or positive microbiologic results. A universal vital assessment score of >4 was associated with all-cause mortality (hazard ratio, 14.5 [95% confidence interval, 5–42.7]).

**Conclusions:**

The 40-day mortality rate among Ugandan patients with solid tumors who developed chemotherapy-associated febrile illness was high, and few had an identified source of infection. Tuberculosis and bacterial bloodstream infections were the leading diagnoses associated with fever. Tuberculosis should be included in the differential diagnosis for patients who develop fever after receiving chemotherapy in tuberculosis-endemic settings, regardless of HIV serostatus.

By 2030, more than 1.28 million new cancer cases and 970 000 cancer-related deaths will occur in sub-Saharan Africa (sSA) annually [1]. Infections are a leading cause of disease and death in patients with cancer receiving chemotherapy. While the microbiology of chemotherapy-related infections in the United States and Europe is well described, the microbiology of these infections in sSA is poorly understood. However, the microbiology of community-acquired febrile illness differ significantly between sSA and the United States or Europe [2–4]. For example, tuberculosis and gram-negative organisms, including *Salmonella* species, frequently cause bloodstream infections (BSIs) throughout sSA [2, 4].

In sSA, up to 35% of patients with a cancer diagnosis are living with human immunodeficiency virus (HIV) [5]. Persons living with HIV (PLWH) may be at high risk of developing opportunistic infections during cancer treatment [6, 7]. In sSA, the prevalence of latent tuberculosis infection (LTBI) is 26%–33%; tuberculosis-related BSIs account for 25%–30% of community-acquired sepsis cases among PLWH [8–10]. Studies from the United States and Europe show that patients with cancer who originated in tuberculosis-endemic settings are at increased risk of developing active tuberculosis after receiving chemotherapy, regardless of HIV serostatus [11–13]. However, the role of tuberculosis as a cause of febrile illness among patients receiving chemotherapy in sSA is unknown.

In Uganda, patients with cancer who develop febrile illness are 2–3 times more likely to die than their counterparts in the United States or Europe [14–17]. Identifying the microbiology of febrile illness among patients receiving chemotherapy is critical for improving outcomes, but access to microbiology laboratories is limited in many countries throughout sSA and microbiology testing is often prohibitively expensive [18]. Thus, clinicians rely on guidelines to direct empiric antibiotic management of suspected infections. Current international guidelines for managing chemotherapy-related infections were developed in the United States and Europe and may not accurately reflect the microbiology and antimicrobial resistance patterns of infections occurring in sSA [19, 20]. Accordingly, our primary study objective was to describe the microbiology of febrile illness among patients with solid tumors receiving systemic chemotherapy at the Uganda Cancer Institute (UCI). Our secondary objective was to determine the predictors of mortality for patients who developed febrile illness within 30 days of receiving systemic chemotherapy.

## METHODS

### Study Design

We conducted a prospective cohort study of adult patients with solid tumors who developed fever within 30 days of receiving chemotherapy and were admitted to the UCI from September 2019 to June 2022.

### Study Setting and Populations

The UCI is a national cancer referral hospital located in Kampala, Uganda. More than 6000 patients with newly diagnosed cancer are treated at the UCI annually; 80% have solid tumors [5]. Adults with solid tumors typically receive chemotherapy in the outpatient infusion clinic and may be admitted to either the Solid Tumor Centre or a private ward if they develop an associated acute illness. Patients admitted to the private ward pay an additional daily charge for semiprivate rooms and a higher nurse-to-patient ratio. Medical officers, supervised by fellowship-trained oncologists, provide inpatient care. Per UCI guidelines, antimicrobial prophylaxis with ciprofloxacin, fluconazole, and acyclovir is recommended for patients who are expected to be neutropenic for >7 days. Antibiotics that are on formulary and in stock in the UCI pharmacy are available without charge.

### Participant Recruitment

We consecutively enrolled participants admitted to the Solid Tumor Centre or a private ward. We included patients ≥18 years of age who had a documented axillary temperature of ≥37.5°C and had received systemic chemotherapy within the prior 30 days. We excluded patients who tested positive for severe acute respiratory syndrome coronavirus 2 using on-site polymerase chain reaction–based testing. For participants with >1 hospital admission during the study period, we evaluated each admission independently. We determined HIV status based on the UCI medical record, per the UCI standard of care.

### Data Collection

Before study enrollment, UCI clinical staff obtained blood for bacterial cultures and malaria rapid diagnostic testing (RDT). They obtained urine for urinary lipoarabinomannan (LAM) testing when the test was in stock. PLWH also underwent blood testing to determine the CD4^+^ T-cell count and serum cryptococcal antigen (CrAg) result.

On study enrollment, study staff reviewed the participant's medical records and completed a history and physical examination. We prospectively followed up participants until hospital discharge or death. We recorded all microbiologic results and prescribed antibiotics. For participants who survived to discharge, we determined the vital status 40 days after fever onset with a follow-up telephone call. We used the severe adverse events form to determine the cause of death. This standardized form is required by the UCI Regulatory and Ethics Committee and includes a narrative of the participant's death. It was completed based on a review of the medical record, discussion with the treating medical team, and discussion with participant's family members. All clinical care was provided by the inpatient clinical team independent of the study team.

### Laboratory Procedures

The Makerere University Microbiology Laboratory in the Department of Medical Microbiology (Kampala, Uganda), accredited through the College of American Pathologists, provided blood cultures, malaria RDT, and serum CrAg and urinary LAM testing. For malaria RDT and serum CrAg testing, we used lateral flow assays. For urinary LAM testing, we used the AlereLAM assay (Alere, USA) and considered a result of 1+ to be positive. The Infectious Diseases Institute (Kampala, Uganda), accredited through Clinical Laboratory Improvement Amendments, determined CD4^+^ T-cell counts. The UCI laboratory provided the results for complete blood cell counts and complete metabolic panels.

### Blood Culture Testing

Because there was a shortage of blood culture bottles in Uganda, we inoculated 1 aerobic culture per participant. The cultures were incubated in a BACTEC 9120 blood culture system (Becton-Dickinson). We used the Kirby-Bauer disc diffusion method to determine drug susceptibilities, according to the Clinical and Laboratory Standards Institute standards [17]. We defined contaminants as coagulase-negative staphylococci, *Bacillus* spp, *Micrococcus* spp, or *Corynebacterium* spp. We considered Enterobacterales and *Enterococcus* to be multidrug resistant (MDR) if they were nonsusceptible to ≥1 agent in ≥3 antimicrobial categories [21].

### Statistical Analysis

We used descriptive statistics to summarize categorical variables as frequencies and percentages and continuous variables as medians with interquartile ranges (IQRs). We determined associations between HIV serostatus and positive microbiology testing using Cox proportional hazards models or Fisher's exact test, as appropriate. To risk-stratify participants, we calculated the universal vital assessment (UVA) score based on the participant's temperature, heart and respiratory rates, systolic blood pressure, oxygen saturation, Glasgow coma score, and HIV serostatus at the time of study enrollment [22].

We used Cox proportional hazards models adjusted for age and sex to investigate the association between all-cause mortality rate and positive microbiologic test results. For tests in which fewer than 4 deaths occurred, we applied Fisher's exact test. For categorical variables, we also used Kaplan-Meier curves and log-rank tests to determine group differences. For the 5 participants with 2 febrile episodes, we used both episodes in all analyses without correction for repeated observations. However, we conducted sensitivity analyses using mixed effects Cox models, adjusted for age and sex, to confirm that the findings were not affected [23]. We considered differences to be statistically significant at *P* < .05. We entered data in Research Electronic Data Capture (REDCap) using double data entry and completed our analysis using R software (R Foundation for Statistical Computing, version 4.3.1).

### Patient Consent Statement

The UCI Regulatory and Ethics Committee (no. 15–2018), the Uganda National Council on Science and Technology (no. 2591), the University of Virginia Institutional Review Board (no. 21372), and the Fred Hutchinson Cancer Center Institutional Review Board (no. 10377A) approved the study. All participants provided written informed consent; for those unable to consent, written consent was obtained from their next of kin.

## RESULTS

### Participant Characteristics

Among the 100 study participants, 1 was lost to follow-up and 99 were included in the analysis. Of these 99 participants, 67 (68%) were female and 24 (24%) were PLWH (Table 1). Among the 24 PLWH, CD4^+^ T-cell counts were determined in 21 (88%). The most frequently encountered cancers were breast (30%), prostate (11%), and hepatobiliary (7%) cancer. Supplementary Figure 1 shows HIV status by cancer type.

**Table 1.** Characteristics of Adult Inpatients With Solid Tumors Who Developed Fever Within 30 Days of Receiving Chemotherapy, September 2019 to June 2022

Characteristic	Patients, No. (%)^a^ (n = 99)
Demographics	
Age, median (IQR), y	42 (33–57)
Female sex	67 (68)
Cancer type	
Breast	30 (30)
Prostate	11 (11)
Gastrointestinal	10 (10)
Head and neck	8 (8)
Hepatobiliary	7 (7)
Ovarian	7 (7)
Kaposi sarcoma	6 (6)
Other sarcoma	6 (6)
Choriocarcinoma	5 (5)
Cervical	4 (4)
Genitourinary	3 (3)
Other	2 (2)
Comorbid conditions	
Hypertension	9 (9)
Diabetes	4 (4)
Liver disease	2 (2)
HIV	
Positive status	24 (24)
Current ART	24 (100)
Time since diagnosis, median (IQR), y	7 (2–13)
CD4^+^ T-cell count, median (IQR), cells/µL (n = 21)	136 (68–300)

Abbreviations: ART, antiretroviral therapy; HIV, human immunodeficiency virus; IQR, interquartile range.

^a^Data represents no. (%) of patients unless otherwise indicated.

### Febrile Episodes

Among the 99 study participants there were 104 febrile episodes; 94 participants had 1 febrile episode and 5 participants had 2 febrile episodes. The median (IQR) time from the last dose of chemotherapy until fever onset was 11 (6–21) days. The median (IQR) absolute neutrophil count at the time of fever onset was 0.95/µL (0.25­–5.06/µL); 40 (38%) of the 104 episodes occurred in the setting of neutropenia (absolute neutrophil count <0.5/µL) (Table 2). The median UVA score at time of enrollment was 0.5.

**Table 2.** Clinical and Laboratory Findings in Adult Inpatients With Solid Tumors Who Developed Fever Within 30 Days of Receiving Chemotherapy, September 2019 to June 2022

Clinical or Laboratory Finding	Median (IQR) Value or No. (%) of -Febrile Episodes (N = 104)
Vital signs, median (IQR)	
Axillary temperature, °C	38.1 (37.9–38.5)
Pulse rate, beats/min	110 (103–119)
Systolic blood pressure, mm Hg	121 (107–131)
Diastolic blood pressure, mm Hg	80 (65–90)
Oxygen saturation, %	96 (94–97)
Required oxygen, no. (%)	21 (20)
Abnormal findings at physical examination	
Any abnormal findings	93 (89)
Head/neck	51 (49)
Respiratory	44 (44)
Cardiac	35 (34)
Gastrointestinal	31 (30)
Skin/soft-tissue	36 (35)
Neurologic	3 (3)
Lymphatic	33 (32)
Other	11 (11)
UVA score, no. (%)	
<2	67 (64)
2–4	29 (28)
>4	8 (8)
Laboratory values, median (IQR)	
Blood cell counts, cells/µL	
White blood cells	2.04 (0.89–7.17)
Neutrophils (ANC)	0.95 (0.25–5.06)
Lymphocytes	0.74 (0.35–1.27)
Platelets/µL	129 (59–202)
Hemoglobin, g/dL	8.9 (7.7–10.95)
Creatinine, µmol/L	58.35 (45.88–80.23)

Abbreviations: ANC, absolute neutrophil count; IQR, interquartile range; UVA, universal vital assessment.

Of the 104 febrile episodes, 83 (80%) had a clinically suspected source of febrile illness (Supplementary Table 1). A central catheter was present in episodes 3 (3%) and a urinary catheter in 2 (2%). Table 2 shows clinical characteristics at the time of study enrollment.

### Microbiology of Febrile Illness

Among the 104 febrile episodes, 30 (29%) had ≥1 positive microbiology test result (Table 3). Of these, 27 (90%) were positive for 1 pathogen and 3 (10%) for 2 pathogens. Among the 3 febrile episodes with >1 pathogen, 2 had a positive blood culture and a positive urinary LAM result, and 1 had a positive malaria RDT and a positive urinary LAM result. Blood culture results were available for 97 (97%) of 104 febrile episodes; during 39 (40%) of these 97 episodes, participants were receiving antibiotics at the time of culture collection. Of these febrile episodes, 12 (12%) of 97 were positive for ≥1 bacterial pathogen. The most common causes of bacteremia were *Escherichia coli*, *Klebsiella pneumoniae*, and *Enterococcus* spp. There was no difference in the prevalence of positive blood cultures between participants with or without HIV (12% vs 13%; *P* = .23 [Fisher's exact test]).

**Table 3.** Positive Microbiologic Results Among 104 Febrile Episodes in Patients With Solid Tumors who Developed Fever Within 30 Days of Receiving Chemotherapy, September 2019 to June 2022

Results	Patients, No. (%)			*P* Value^a^
	HIV Seronegative (n = 73)	HIV Seropositive (n = 24)	Total (n = 97)	
Blood culture results, no. (%)				
Any positive blood culture^b^	9 (12)	3 (13)^c^	12 (12)	.23
*Escherichia coli*	5 (7)	1 (4)	6 (6)	
*Klebsiella pneumoniae*	2 (3)	2 (9)	4 (4)	
*Enterococcus* spp	1 (1)	1 (4)	2 (2)	
*Staphylococcus* spp	1 (1)	0 (0)	1 (1)	
Other microbiologic results				
Malaria RDT (n = 97 tested)	8/75 (11)	0/22 (0)	8/97 (8)	.19
Urinary LAM (n = 62 tested)	8/44 (18)	4/18 (22)	12/62 (19)	.09
Serum CrAg (n = 12 tested)^d^	…	1/12 (8)	1/12 (8)	…

Abbreviations: CrAg, cryptococcal antigen; HIV, human immunodeficiency virus; LAM, lipoarabinomannan; RDT, rapid diagnostic test.

^a^ *P* values based on Fisher's exact test.

^b^Five cultures were positive for coagulase-negative *Staphylococcus*, 1 for *Micrococcus* spp, 1 for gram-positive rods, and 1 for *Corynebacterium* spp, which were considered contaminants.

^c^One HIV-positive participant experienced polymicrobial bacteremia.

Malaria RDT results were available for 97 (93%) of 104 febrile episodes. Of these, 8 (8%) of 97 were positive. There was no difference in the prevalence of malaria between participants with or without HIV (0% vs 8%; *P* = .19 [Fisher's exact test]). Urinary LAM results were available for 62 (60%) of 104 febrile episodes. Of these, 12 (19%) of 62 were positive. There was no difference in the prevalence of positive urinary LAM results between participants with or without HIV (22% vs 19%; *P* = .09 [Fisher's exact test]). The median (IQR) lymphocyte count was lower among patients with a positive than among those with a negative urinary LAM result, 0.40/µL (0.21–0.77/µL) versus 0.66/µL (0.19–3.45/µL), although this difference was not statistically significant (*P* = .33 [Wilcoxon rank sum test]). Among the 24 PLWH, 12 (50%) had serum CrAg results available, 1 (8%) of which was positive.

### Antimicrobial Resistance

Among the 10 Enterobacterales isolated, 8 (80%) were MDR organisms. Of the isolates tested, 8 (88%) of 9 were resistant to ceftriaxone, 2 (25%) of 8 were resistant to carbapenems, and 3 (38%) of 8 were resistant to chloramphenicol. Among the 2 *Enterococcus* spp isolated, 1 was penicillin susceptible and 1 was resistant to penicillin, ampicillin, vancomycin, linezolid, and chloramphenicol.

### Antimicrobial Management

In 18 (17%) of 104 febrile episodes, participants reported taking antibiotics within the prior 30 days. In 18 (17%) of 104 febrile episodes, participants were already prescribed antibacterials at the time of fever onset (Supplementary Figure 2). In 95 (91%) of 104 febrile episodes, participants had ≥1 antibiotic prescribed on or after the day of fever onset. The median (IQR) time from fever onset to a new antibiotic prescription was 0 (0–1) days. Supplementary Figure 3 shows antibiotics prescribed after fever detection. All 12 participants (100%) with a BSI had empiric antibiotics prescribed on the day of blood culture collection; 6 of 12 (50%) had an isolate that was susceptible to the empiric antibiotic (Supplementary Table 2). Of the 6 with a nonsusceptible isolate, 2 (33%) had an appropriate antibiotic subsequently prescribed. All 8 participants who received appropriate antibiotics were alive at 40 days; all 4 participants who never received an appropriate antibiotic died.

Of the 12 participants with a positive urinary LAM result, 9 (75%) were started on antituberculosis therapy, 1 (8%) died before starting therapy, and 2 (17%) were lost to follow-up. All 8 participants with malaria were treated with antimalarial therapy. The participant with a positive CrAg result received amphotericin B.

### Patient Outcomes

**Figure 1.**
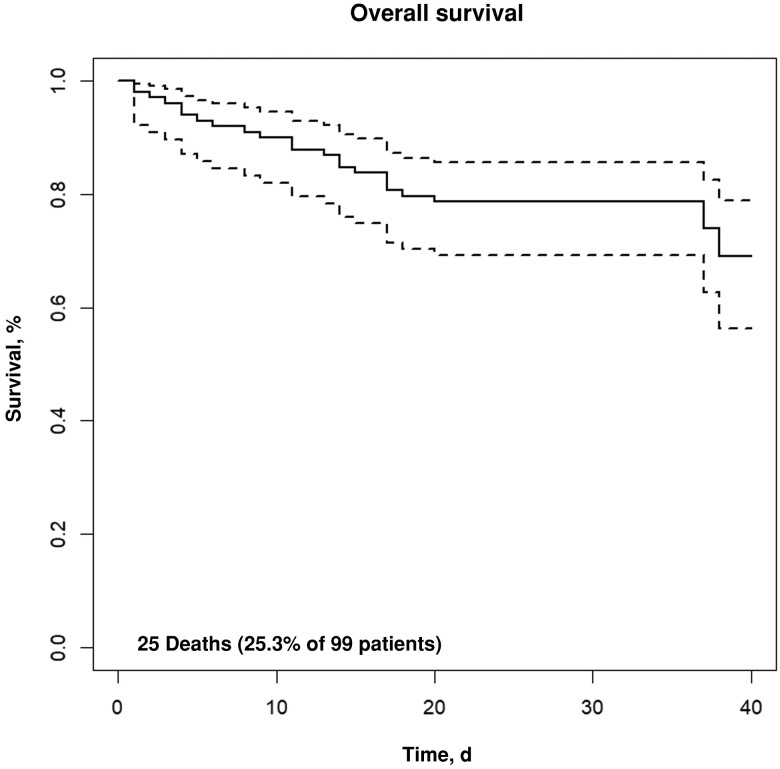
Kaplan-Meier curve of patient survival at 40 days after fever onset for patients at the Uganda Cancer Institute who developed fever within 30 days of receiving chemotherapy. Dashed lines represent the 95% confidence interval of the Kaplan-Meier curve.

Of the 99 participants, 25 (25%) died within 40 days after fever onset (Figure 1). Of these 25 patients, 17 (68%) died in the hospital and 8 (32%) died after hospital discharge. Among the 25 who died, 18 (72%) had a documented cause of death. Eleven (61%) of the 18 deaths were attributed to infection, 3 (17%) to cancer, 2 (11%) to hemorrhage, and 2 (11%) to other causes (Supplementary Table 3).

**Figure 2.**
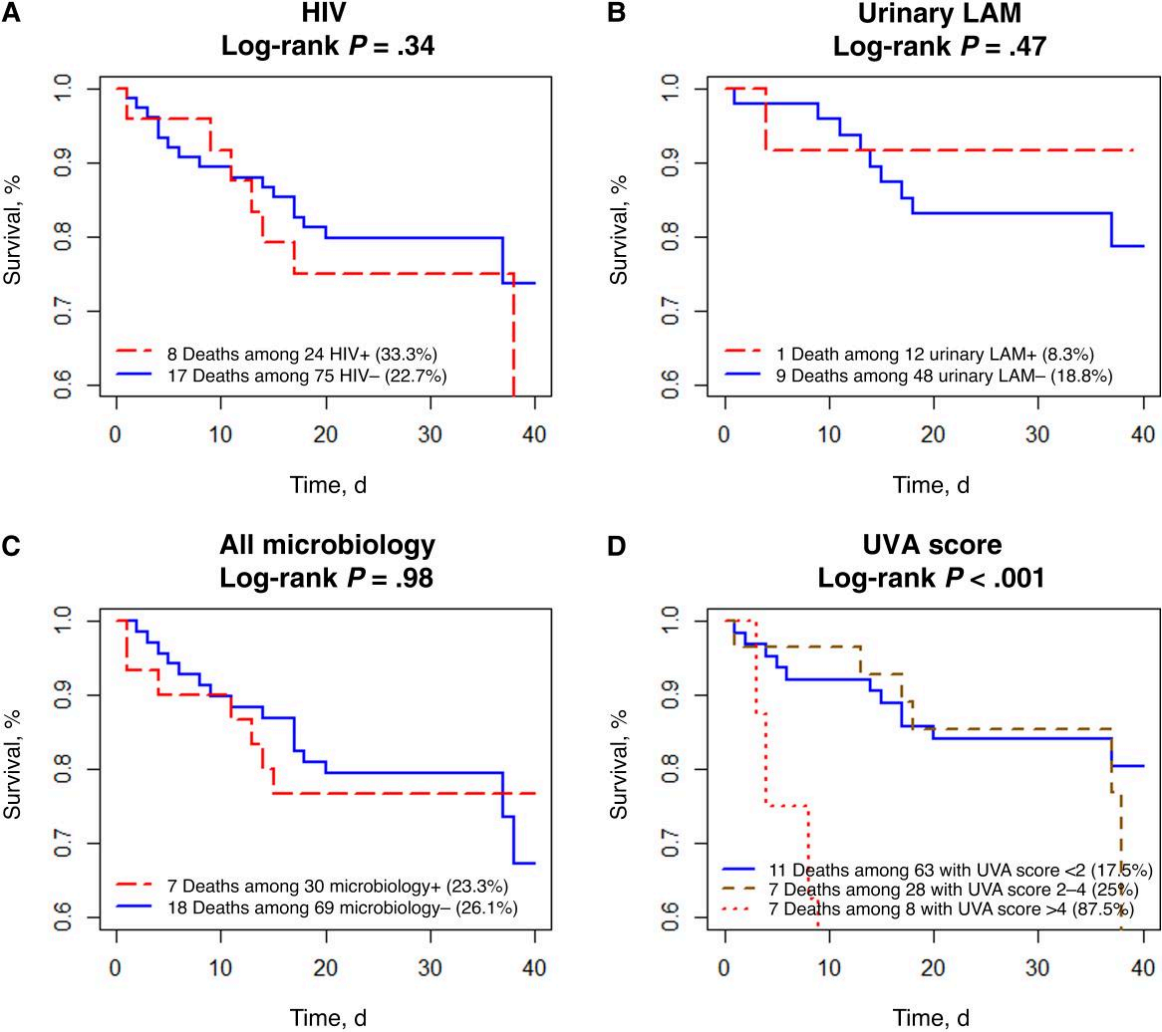
Kaplan-Meier estimates with log-rank test of 40-day survival rate based on human immunodeficiency virus (HIV) status (*A*), urinary lipoarabinomannan (LAM) positivity (*B*), positive microbiologic (microbiology+) results (*C*), and universal vital assessment (UVA) score (*D*). Results for “all microbiology” were defined as positive if any of the following results was positive: blood culture, urinary LAM, malaria rapid diagnostic test, or serum cryptococcal antigen.

After adjustment for age and sex, there was no association between the 40-day mortality rate and HIV status, presence of neutropenia, blood culture positivity, or positive microbiology test result (defined as ≥1 of the following results positive: blood culture, urinary LAM, malaria RDT, or serum CrAg) (Table 4, Figure 2, and Supplementary Table 4). There was also no association between participant death and malaria positivity (*P* > .99 [Fisher's exact test]) or urinary LAM positivity (*P* = .67 [Fisher's exact test]). A UVA score >4 was associated with increased mortality rate (hazard ratio, 14.5 [95% confidence interval, 5­–42.7]; *P* < .001 [Cox model]) (Table 4 and Figure 2).

**Table 4.** Cox Regression Analysis of the Association Between Participant Characteristics and 40-Day All-Cause Mortality Rate in Adult Patients With Solid Tumors Who Developed Fever Within 30 Days of Receiving Chemotherapy, Adjusted for Age and Sex

Characteristic	Hazard Ratio (95% CI)	*P* Value
HIV status	1.64 (.69–3.9)	.26
Neutropenia	0.98 (.41–2.34)	.97
Positive blood culture	1.85 (.61–5.6)	.27
Positive microbiologic result^a^	0.8 (.36–1.83)	.61
UVA score^b^		
<2	…	…
2–4	1.57 (.6–4.14)	.35
>4	14.5 (5–42.7)	<.001

Abbreviations: CI, confidence interval; HIV, human immunodeficiency virus; UVA, universal vital assessment.

^a^Includes ≥1 positive result among the following studies: blood culture, urinary lipoarabinomannan, malaria rapid diagnostic test, and serum cryptococcal antigen.

^b^UVA score based on temperature, heart and respiratory rates, systolic blood pressure, oxygen saturation, Glasgow coma scale score, and HIV serostatus at the time of study enrollment.

## DISCUSSION

In this study, we prospectively evaluated the microbiology of febrile illness among adults with solid tumors who were admitted to the UCI and developed fever within 30 days after receiving chemotherapy. We found that most patients had no identified infectious cause of febrile illness. Tuberculosis and bacterial BSIs were the most frequently identified causes of infection. There was no significant difference in the microbiology of febrile illness based on HIV status. When compared with similar cohorts in the United States and Europe, the 40-day case fatality ratio (CFR) of 25% was high. We did not find a significant association between all-cause mortality rate and HIV status, presence of neutropenia, or positive microbiologic findings. However, a UVA score >4 was associated with increased all-cause mortality rate.

Only 29% of participants had a microbiologically diagnosed cause of febrile illness. This is similar to studies from the United States, in which 20%–30% of patients with cancer who develop neutropenic fever have an identified source of infection [19]. In our study, tuberculosis was the most frequently identified cause of febrile illness. Two meta-analyses estimated that the incidence of active tuberculosis among patients with solid tumors is 2–17 times greater than that of the general population [24, 25]. However, only 3 studies included in these meta-analyses were conducted in an African country: 2 in South Africa and 1 in Algeria. Since most of the participants in these meta-analyses were from low-tuberculosis endemicity settings, those who developed active tuberculosis likely experienced LTBI reactivation.

Patients living in high-tuberculosis endemicity settings may be at even higher risk of developing active tuberculosis owing to increased susceptibility to new infections. Consensus recommendation advise that healthcare providers consider prechemotherapy LTBI screening only for those with high-risk cancers (eg, hematologic, lung, or head-and-neck cancer) [26]. However, in Uganda, where up to 40% of the general population has LTBI, screening and treating patients with lower-risk cancers could decrease morbidity and mortality rates. We did not find a significant difference in tuberculosis diagnosis by HIV status. Thus, tuberculosis should be considered a potential cause of febrile illness for patients receiving chemotherapy in a high-tuberculosis endemicity setting, irrespective of HIV status.

Urinary LAM is a first-line point-of-care test for diagnosing systemic tuberculosis among PLWH. Our study was among the first to use urinary LAM testing among patients with cancer. For the AlereLAM assay, the pooled sensitivity ranges from 18% for those who are HIV seronegative to 42% for those who are HIV seropositive [27]. The highest sensitivity occurs among those with a CD4^+^ T-cell count <200/µL. Because CD4^+^ T-cell counts were available only for PLWH, we could not evaluate the association between these counts and LAM positivity in our study. However, the median lymphocyte count was lower for those who were LAM positive than to those who were LAM negative, although this difference was not statistically significant.

Recently developed second-generation urinary LAM tests have a sensitivity of up to 70% for detecting active tuberculosis among PLWH [28]. However, little is known about the sensitivity of urinary LAM among non–HIV-immunocompromised individuals [29]. While it is possible that some urinary LAM results were false-positives, the AlereLAM assay has a 96%–98% specificity [27]. Thus, in the setting of high LTBI prevalence and participant immunosuppression, false-positives are less likely. Participants with a positive LAM result were referred to the Mulago Hospital tuberculosis ward, and we did not have access to the results of any subsequent imaging, sputum GeneXpert testing, or sputum culture testing. Given the high mortality rate among patients with cancer who develop tuberculosis, prompt diagnosis and treatment initiation could significantly improve outcomes. Further research is needed to determine whether urinary LAM should be used as a standard test for diagnosing tuberculosis among patients with cancer.

Among enrolled participants, only 12% had a documented bacterial cause of febrile illness. However, 40% were receiving an antibiotic when blood cultures were collected, and we were able to obtain only 1 aerobic blood culture per participant. Thus, our study likely underestimated the true prevalence of bacteremia [30, 31]. The most frequently isolated bacterial pathogens were enteric organisms, including *E coli, Klebsiella,* and *Enterococcus*. Similarly, we previously found that 80% of UCI patients with hematologic cancers who developed a bacterial BSI were infected with an enteric gram-negative organism [16]. Eighty percent of the gram-negative organisms isolated in our study were MDR, which reflects the crisis of increasing MDR gram-negative BSIs in sSA [32–34]. There is limited availability of broad-spectrum antibiotics throughout much of sSA, and available antibiotics are often prohibitively expensive (eg, carbapenems and colistin) and/or associated with significant toxic effects (eg, bone marrow suppression with chloramphenicol and renal toxicity with colistin). Given the high CFR that occurs among patients with cancer who develop MDR bacterial BSIs [35, 36], our findings emphasize the critical importance of ensuring access to broad-spectrum antibiotics in sSA. It is also important to develop infection treatment guidelines that account for local antibiotic susceptibilities and locally available antibiotics.

The prevalence of HIV in our study was 24%, which is similar to that in a previous study at UCI [5]. We found no differences in the microbiology of febrile illness based on HIV status, although our study was underpowered to detect such a difference. Several small studies suggest that PLWH may experience higher rates of chemotherapy-related bone marrow suppression and treatment-induced neutropenia [37, 38]. Other studies show that persons with poorly controlled HIV are at increased risk of experiencing an opportunistic infection within the first 6 months after chemotherapy initiation [39]. Understanding the associations between HIV and chemotherapy-related infections is critical for developing infection management guidelines for patients receiving chemotherapy in HIV-endemic settings. This is especially important in countries like Uganda where there is limited access to clinical microbiology laboratories and clinicians rely heavily on standardized antibiotic treatment guidelines.

While the 25% 40-day CFR in our cohort was high, it is similar to the 7%–22% CFR for patients with sepsis in Uganda [40–42]. Infection was the most frequently identified cause of death. There was no significant difference in mortality rate by HIV status, presence of neutropenia, or a detected infection, which may reflect our relatively small sample size. Studies show that a shoter time to antibiotics is associated with improved outcome, for patients with sepsis. In our study, 90% of participants had a new antibiotic prescribed on or after the day fever was detected. However, for those with bacteremia, only 50% had an initial antibiotic prescribed that treated the isolated bacteria. We also found that not all prescribed antibiotics were documented as being administered, which could be related to antibiotic stockouts. Given the association between elevated UVA score and mortality, the UVA score may be a useful tool to risk-stratify patients admitted with postchemotherapy fever at UCI.

Our study had limitations. We prospectively enrolled a small cohort from a single cancer center in Uganda. While we believe our findings can be extrapolated to similar patients hospitalized at UCI, our results may not be correlated with the microbiology of febrile illness that occurs in other cancer centers in sSA. Since our study enrolled only hospitalized patients with febrile illness, additional studies are needed to define the prevalence, microbiology, and outcomes among outpatients with febrile illness. Our study relied on malaria RDTs without corresponding malaria microscopy, so it is difficult to determine which participants had active malaria. Owing to the limited number of pathogens that can be detected using standard microbiologic tests and the laboratory test stockouts that occurred, we likely underestimated the prevalence of infection-related causes of febrile illness. Molecular tests, including whole-genome sequencing, may be useful for identifying additional pathogens that could not be detected using standard methods [43]. Finally, our study took place during the coronavirus disease 2019 pandemic, which could have altered the microbiology of febrile illness.

In conclusion, nearly one-third of UCI adult inpatients with solid tumors who developed febrile illness within 30 days of receiving chemotherapy had an identified cause of fever. Tuberculosis and gram-negative BSI were the most frequently identified causes. These findings emphasize the need to better understand the incidence and risk factors for development of active tuberculosis among patients receiving chemotherapy in high-endemicity tuberculosis settings. This will inform strategies to prevent and treat tuberculosis among patients receiving cancer treatment. Meanwhile, clinicians in sSA should consider tuberculosis as a potential cause of chemotherapy-related illness regardless of HIV serostatus. They should also consider MDR bacteria as a potential cause of febrile illness when selecting empiric antibiotics. Given the high 40-day CFR, additional studies are needed to clarify the infectious causes of febrile illness among patients receiving cancer treatment in sSA. This will support the development of infection management guidelines that adequately address local causes of febrile illness in African populations.

## Supplementary Data

Supplementary materials are available at *Open Forum Infectious Diseases* online. Consisting of data provided by the authors to benefit the reader, the posted materials are not copyedited and are the sole responsibility of the authors, so questions or comments should be addressed to the corresponding author.

## Supplementary Material

ofad508_Supplementary_Data
