# Supplementary material for: Etiology of Fever and Associated Outcomes Among Adults Receiving Chemotherapy for the Treatment of Solid Tumors in Uganda
Source: Open Forum Infect Dis. 2023 Oct 12;10(11):ofad508. doi: 10.1093/ofid/ofad508 (PMC10633783; doi:10.1093/ofid/ofad508)
Supplement: ofad508_Supplementary_Data [file ofad508_supplementary_data.zip › Supplementary Table 2.docx]

| **Supplementary Table 2.** Antimicrobial management and associated outcome for patients with bloodstream infections at UCI | | | | | |
| --- | --- | --- | --- | --- | --- |
|  | **Blood culture result** | **Antibiotic resistance^a^** | **Antibiotics prescribed^b^** | **Antibiotics administered^b^** | **Death^c^** |
| **1** | *Staphylococcus spp.* |  | Flucloxacillin/amoxicillin (d1-2) | Flucloxacillin/amoxicillin (d1) | N |
| **2** | *Klebsiella pneumoniae* | ESBL  Chloramphenicol resistant | Piperacillin/tazobactam (d1-2) | Piperacillin/tazobactam (d1-2) | Y (d2) |
| **3** | *Klebsiella pneumoniae* | ESBL  Chloramphenicol resistant | Piperacillin/tazobactam (d1-2) | Piperacillin/tazobactam (d1-2)  Metronidazole (d2) | Y (d2) |
| **4** | *Klebsiella pneumoniae* | ESBL | Meropenem (d1-5)  Moxifloxacin (d1-5) | Meropenem (d1-4)  Moxifloxacin (d3-4) | N |
| **5** | *Escherichia coli* |  | Ampicillin (d1-5) Piperacillin/tazobactam (d1-5) | Piperacillin/tazobactam (d1-4) | N |
| **6** | *Escherichia coli* | ESBL | Piperacillin/tazobactam (d1-4) Ceftriaxone (d1-4)  Imipenem (d2-6) | Moxifloxacin (d2-4; d6-7)  Ceftriaxone/sulbactam (d2-4)  Imipenem (d2-5)  Metronidazole (d6-8) | N |
| **7** | *Escherichia coli* | ESBL  Chloramphenicol resistant | Moxifloxacin (d1-6)  Imipenem (d7-d10) | Moxifloxacin (d1-5) | N |
| **8** | *Escherichia coli* | ESBL | Piperacillin/tazobactam (d1-5) Meropenem (d1-6) | Piperacillin/tazobactam (d1-5)  Meropenem (d2-5) | N |
| **9** | *Enterococcus spp.* |  | Moxifloxacin (d1-6) | Piperacillin/tazobactam (d8-10) | Y (d10) |
| **10** | *Escherichia coli* | ESBL | Moxifloxacin (d1-2)  Meropenem (d3-7) | Moxifloxacin (d1)  Meropenem (d3-7) | N |
| **11** | *Klebsiella pneumoniae* |  | Piperacillin/tazobactam (d1-6)  Gentamicin (d1-2)  Meropenem (d5-12)🡪 Gentamicin (d12-16)🡪 Ceftriaxone/sulbactam (d12-16) | Piperacillin/tazobactam (d4-6)  Meropenem (d5-9) | N |
| **12** | *Escherichia coli* | ESBL  Intermediate sensitivity to imipenem | Ceftriaxone/sulbactam (d1-5)  Ornidazole (d3-13)  Piperacillin/tazobactam (d6-12)  Chloramphenicol (d11-12) | Piperacillin/tazobactam (d9-13)  Chloramphenicol (d12-13) | Y (d13) |
|  | *Enterococcus spp.* | VRE  Ampicillin resistant |  |  |  |
| ^a^ Based on phenotypic resistance. ESBL: extended spectrum beta-lactamase, VRE: vancomycin resistant *Enterococcus*  ^b^Indicates the number of day(s) that that antibiotics were prescribed or administered, starting from the day of fever onset. Prescribed and administered antibiotics as per documentation in the Uganda Cancer Institute paper medical record.  ^c^Numbers indicate the number of days from fever onset until death occurred | | | | | |
