# Supplementary material for: Etiology of Fever and Associated Outcomes Among Adults Receiving Chemotherapy for the Treatment of Solid Tumors in Uganda
Source: Open Forum Infect Dis. 2023 Oct 12;10(11):ofad508. doi: 10.1093/ofid/ofad508 (PMC10633783; doi:10.1093/ofid/ofad508)
Supplement: ofad508_Supplementary_Data [file ofad508_supplementary_data.zip › Supplementary Table 3.docx]

| **Supplementary Table 3.** Causes of death among 25 patients with solid tumors who developed febrile illness within 30 days of receiving chemotherapy at the Uganda Cancer Institute. | | | | | | | | | |
| --- | --- | --- | --- | --- | --- | --- | --- | --- | --- |
|  | **Age** | **Sex** | **Cancer type** | **HIV-status** | **Neutropenic** | **Suspected source of infection** | **Positive microbiology tests** | **Antibiotics appropriate** | **Cause of death** |
| **1** | 46 | Male | Hepatocellular carcinoma | Positive | No | No focus of infection |  |  | Hemorrhage |
| **2** | 54 | Female | Breast cancer | Positive | No | Pneumonia |  |  | Unknown |
| **3** | 41 | Female | Cervical cancer | Positive | No | Infected tumor of the cervix |  |  | Unknown |
| **4** | 46 | Female | Breast cancer | Negative | Yes | Infected breast tumor |  |  | Infection |
| **5** | 52 | Female | Breast cancer | Negative | Yes | Urinary tract infection, mucositis | Malaria | Yes | Unknown |
| **6** | 65 | Male | Prostate cancer | Negative | Yes | Thrush, pneumonia |  |  | Malignancy |
| **7** | 24 | Female | Ovarian | Negative | No | Urinary tract infection, pneumonia |  |  | Malignancy |
| **8** | 38 | Female | Glioblastoma multiforme | Negative | No | Infected feeding tube stoma site |  |  | Pneumonia |
| **9** | 60 | Female | Gastrointestinal stromal tumor | Negative | No | No focus of infection |  |  | Malignancy |
| **10** | 58 | Female | Nasopharyngeal carcinoma | Positive | Yes | Thrush, mucositis | *Klebsiella pneumoniae* bacteremia | No | Hemorrhage |
| **11** | 47 | Female | Breast cancer | Negative | Yes | Gastroenteritis, thrush | *Klebsiella pneumoniae* bacteremia | No | Aspiration pneumonia |
| **12** | 70 | Male | Esophageal carcinoma | Positive | Yes | Thrush, pneumonia | Cryptococcus | Yes | Aspiration pneumonia |
| **13** | 56 | Male | Prostate cancer | Negative | No | No focus of infection |  |  | Aspiration pneumonia, subdural hemorrhage |
| **14** | 37 | Male | Hepatocellular carcinoma | Negative | No | Pneumonia |  |  | Unknown |
| **15** | 26 | Female | Colorectal cancer | Negative | No | Gluteal abscess |  |  | Unknown |
| **16** | 26 | Female | Renal cell carcinoma | Negative | No | No focus of infection |  |  | Pneumonia |
| **17** | 40 | Female | Ovarian cancer | Negative | No | Pneumonia |  |  | Aspiration pneumonia |
| **18** | 64 | Male | Prostate cancer | Negative | No | No focus of infection |  |  | Post-discharge COVID |
| **19** | 58 | Female | Breast cancer | Negative | No | Pneumonia |  |  | Pneumonia |
| **20** | 29 | Female | Kaposi Sarcoma | Positive | No | Esophagitis |  |  | Pneumonia |
| **21** | 56 | Male | Hepatocellular carcinoma | Negative | No | Pneumonia, gastroenteritis |  |  | Unknown |
| **22** | 77 | Female | Breast cancer | Negative | Yes | Mucositis, thrush | *Enterococcus* bacteremia | No | Unknown |
| **23** | 66 | Female | Breast cancer | Negative | Yes | Sinusitis, mucositis, otitis media, thrush, pneumonia |  |  | Unknown |
| **24** | 34 | Female | Parotid pleomorphic adenoma | Negative | Yes | Mucositis, pneumonia, esophagitis | Urinary LAM | No | Acute hypoxic respiratory failure |
| **25** | 26 | Female | Pancreatic cancer | Positive | No | Pneumonia and spontaneous bacterial peritonitis | *Enterococcus* and *Escherichia coli* bacteremia | No | Sepsis |
