# Supplementary material for: Etiology of Fever and Associated Outcomes Among Adults Receiving Chemotherapy for the Treatment of Solid Tumors in Uganda
Source: Open Forum Infect Dis. 2023 Oct 12;10(11):ofad508. doi: 10.1093/ofid/ofad508 (PMC10633783; doi:10.1093/ofid/ofad508)
Supplement: ofad508_Supplementary_Data [file ofad508_supplementary_data.zip › Supplementary Table 4.docx]

| **Supplementary Table 4.** Mixed effects models for Cox regression analysis of the association between participant characteristics and 40-day survival for adult patients with solid tumors who developed febrile illness within 30-days of receiving chemotherapy at the Uganda Cancer Institute, adjusted age and gender. | | | |
| --- | --- | --- | --- |
| **Characteristic** | **Hazard ratio** | **95% CI** | **P-value** |
| HIV status | 1.8 | (0.76, 4.28) | 0.18 |
| Neutropenia | 0.98 | (0.41, 2.39) | 0.97 |
| Positive blood culture | 2.32 | (0.67, 7.99) | 0.18 |
| Positive microbiologic study^a^ | 1.14 | (0.45, 2.88) | 0.78 |
| **UVA score^b^** |  |  |  |
| <2 | --- | --- | --- |
| 2-4 | 1.57 | (0.6, 4.12) | 0.35 |
| >4 | 14.58 | (5, 42.7) | <0.001 |
| ^a^Includes at least one of the following positive studies: blood cultures, urinary lipoarabinomannan, malaria rapid diagnostic test, and serum cryptococcal antigen  ^b^UVA score based on temperature, heart and respiratory rates, systolic blood pressure, oxygen saturation, Glasgow Coma Scale score, and HIV serostatus at the time of study enrollment. | | | |
