# Supplementary material for: Etiology of Fever and Associated Outcomes Among Adults Receiving Chemotherapy for the Treatment of Solid Tumors in Uganda
Source: Open Forum Infect Dis. 2023 Oct 12;10(11):ofad508. doi: 10.1093/ofid/ofad508 (PMC10633783; doi:10.1093/ofid/ofad508)
Supplement: ofad508_Supplementary_Data [file ofad508_supplementary_data.zip › Supplementary Figure 1.docx]

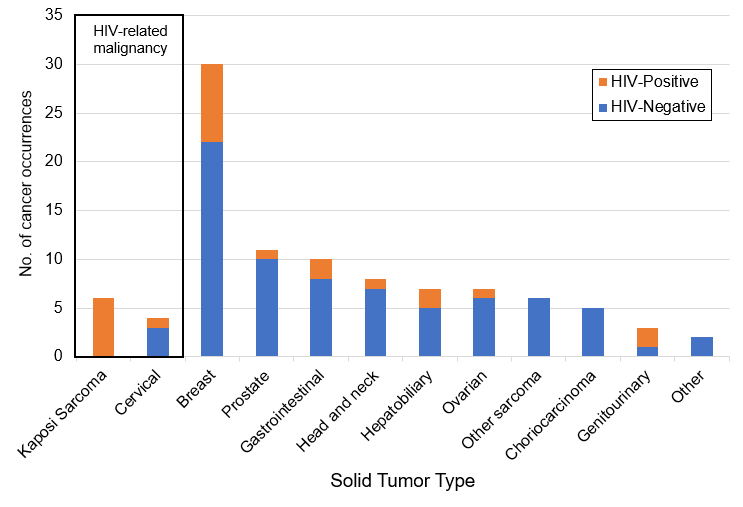


**Supplementary Figure 1**. Type of cancer and associated HIV status among adult inpatients with solid tumors who developed febrile illness within 30 days of receiving chemotherapy at the Uganda Cancer Institute from September 2019-July 2022.
