# Supplementary material for: Etiology of Fever and Associated Outcomes Among Adults Receiving Chemotherapy for the Treatment of Solid Tumors in Uganda
Source: Open Forum Infect Dis. 2023 Oct 12;10(11):ofad508. doi: 10.1093/ofid/ofad508 (PMC10633783; doi:10.1093/ofid/ofad508)
Supplement: ofad508_Supplementary_Data [file ofad508_supplementary_data.zip › Supplementary Figure 2.docx]

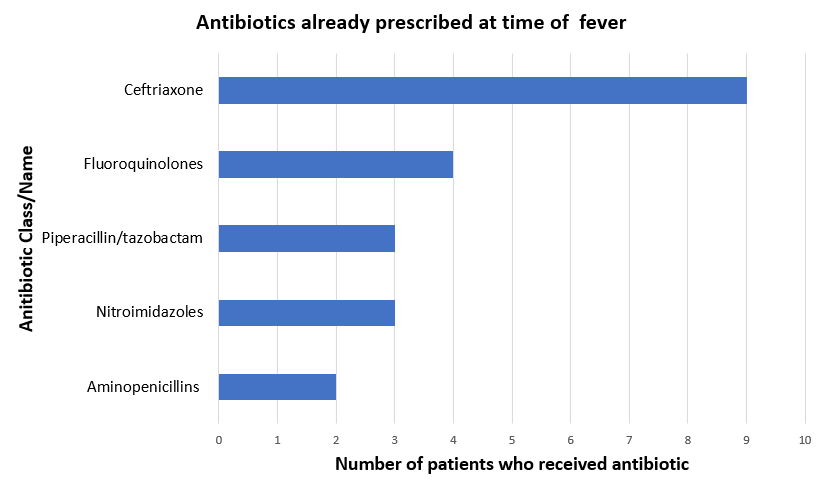


**Supplementary Figure 2**. Antibiotics already prescribed at the time of fever onset for adult inpatients with solid tumors who developed fever within 30-days of chemotherapy at the Uganda Cancer Institute (n=104).^1^

^1^Among the 104 febrile episodes, 18 (17%) had at least one antibiotic already prescribed at fever onset. Of these, 14 (77%) had 1 prescribed antibiotic and 4 (23%) had 2 prescribed antibiotics.
