# Supplementary material for: Etiology of Fever and Associated Outcomes Among Adults Receiving Chemotherapy for the Treatment of Solid Tumors in Uganda
Source: Open Forum Infect Dis. 2023 Oct 12;10(11):ofad508. doi: 10.1093/ofid/ofad508 (PMC10633783; doi:10.1093/ofid/ofad508)
Supplement: ofad508_Supplementary_Data [file ofad508_supplementary_data.zip › Supplementary Figure 3.docx]

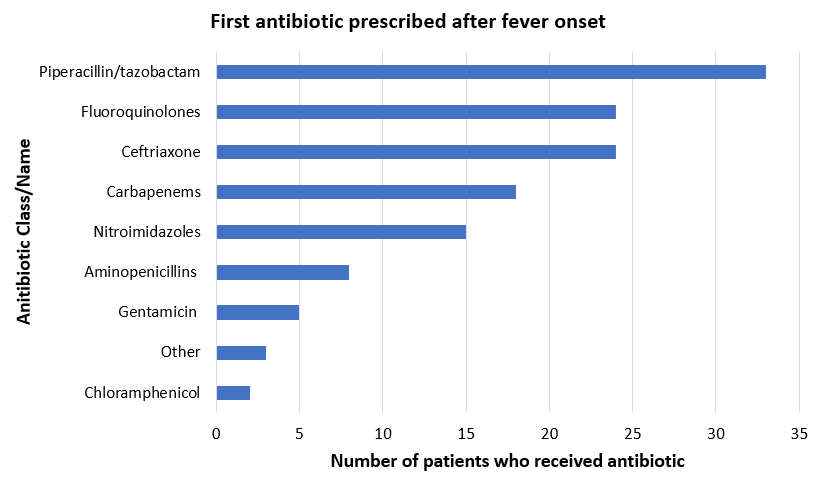


**Supplementary Figure 3**. First antibiotic(s) prescribed on or after the day of fever onset for adult inpatients with solid tumors who developed fever within 30-days of chemotherapy at the Uganda Cancer Institute (n=104).^1^

^1^Among the 104 febrile episodes, 95 (91%) had at least 1 antibiotic prescribed after fever onset. Of these, 61 (64%) had 1 prescribed antibiotic, 31 (33%) had 2 prescribed antibiotics, and 3 (3%) had 3 prescribed antibiotics.
