# Supplementary material for: Etiology of Fever and Associated Outcomes Among Adults Receiving Chemotherapy for the Treatment of Solid Tumors in Uganda
Source: Open Forum Infect Dis. 2023 Oct 12;10(11):ofad508. doi: 10.1093/ofid/ofad508 (PMC10633783; doi:10.1093/ofid/ofad508)
Supplement: ofad508_Supplementary_Data [file ofad508_supplementary_data.zip › Supplementary Table 1.docx]

| **Supplementary Table 1.** Clinically suspected sources of infection for adult inpatients with solid tumors who developed fever within 30 days of receiving chemotherapy, September 2019 – June 2022. | |
| --- | --- |
| **Source of infection^a^** | **n = 104** |
| No localizing source | 21 (20) |
| Localizing source | 83 (80) |
| Mucositis | 40 (38) |
| Thrush | 29 (28) |
| Pneumonia | 22 (21) |
| Abscess | 12 (12) |
| Infected tumor | 12 (12) |
| Gastroenteritis | 11 (11) |
| Urinary tract infection | 11 (11) |
| Esophagitis | 9 (9) |
| Cellulitis | 8 (8) |
| Sinusitis | 5 (5) |
| Intraabdominal infection | 4 (4) |
| Otitis media | 4 (4) |
| Cholangitis | 2 (2) |
| Prostatitis | 2 (2) |
| Hepatitis | 2 (2) |
| Meningitis | 1 (1) |
| Other | 2 (2) |
| ^a^Data are presented as No. (%) unless otherwise indicated  ^b^Numbers add to more than 83, since participants could have more than one suspected source of infection | |
